# Supplementary material for: Ionizing Radiation Activates Mitochondrial Function in Osteoclasts and Causes Bone Loss in Young Adult Male Mice
Source: Int J Mol Sci. 2022 Jan 8;23(2):675. doi: 10.3390/ijms23020675 (PMC8775597; doi:10.3390/ijms23020675)
Supplement: Supplementary file 1 [file ijms-23-00675-s001.zip › ijms-1537786-supplementary.pdf]

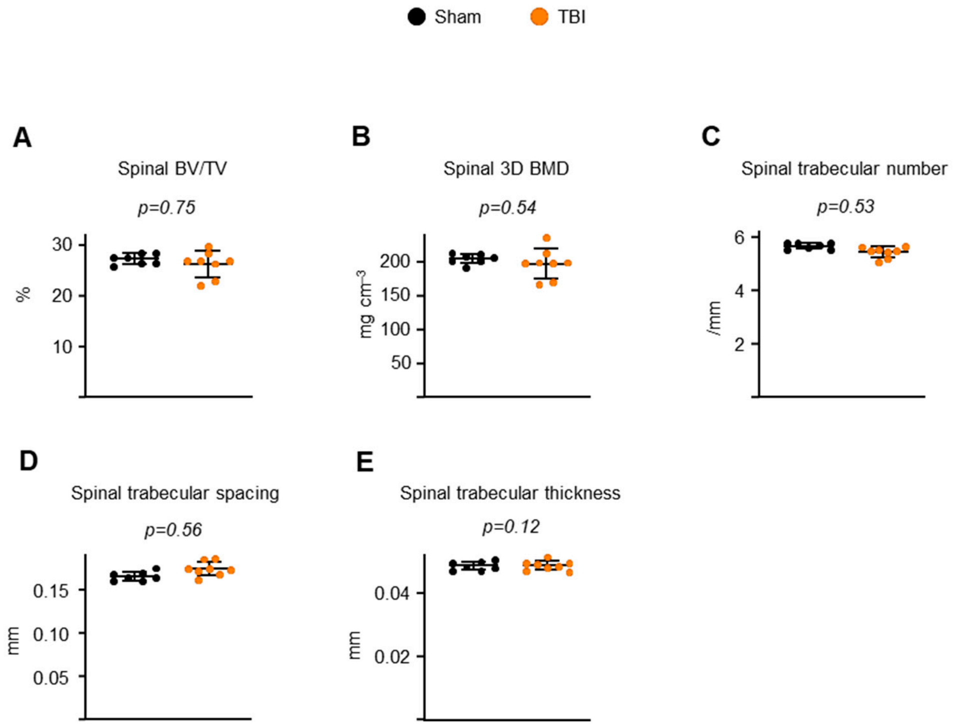

Supplementary Figure S1. Exposure to 0.5 Gy TBI does not alter bone mass in young adult male mice. Quantification of spinal bone mass from 4-month-old male C57BL/6 sham- or IR-exposed mice 4 weeks after irradiation (n=7-8 animals/group). (**A**) Bone volume over tissue volume (BV/TV), (**B**) bone mineral density (BMD), (**C**) trabecular number, (**D**) spacing, and (**E**) thickness of trabecular bone measured in the spine of irradiated mice and sham controls by μCT. Data are presented as ± SD. P values were determined using Student's *t* test.

A

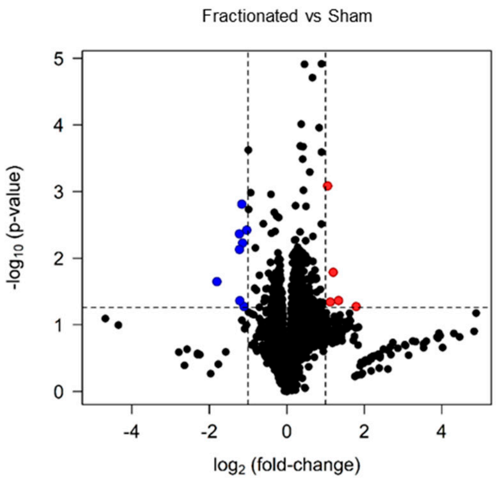

B

Proteins	Fold
**Pex6**	**-1.80644**
S100a8	-1.22432
S100a9	-1.22186
Gm4951	-1.21828
Ctsg	-1.16361

Proteins	Fold
Arhgef7	1.33236
M6	1.196276
Nubp2	1.122101
Banf1	1.053063
Cln6	0.934011

Supplementary Figure S2. Pex6 might be a regulator of TBI exposure in osteoclasts. (**A**-**B**) BMMs were isolated from fractionated TBI mice and sham controls described in Figure 1 and cultured with M-CSF (30 ng/mL) and RANKL (30 ng/mL) for 3 days to make pre-osteoclasts. (**A**) Quantitative analysis of global proteome was performed by label-free tandem mass spectrometry. (**B**) The fold change in total proteins is presented in the Volcano plots for the effects of IR.
